# Fellowships, Grants, & Awards

**Published:** 2004-08

## Cancer Intervention and Surveillance Modeling Network (CISNET)

The Division of Cancer Control and Population Sciences (DCCPS) of the National Cancer Institute (NCI) invites applications from domestic and foreign applicants to support collaborative research using simulation and other modeling techniques to describe the impact of interventions in population-based settings that will shed light on U.S. population-based trends. It is well known that great progress in the war against cancer is possible by the complete use and adequate delivery of existing modalities of cancer control. The primary goals of this research are to determine the impact of cancer control interventions on observed trends in incidence and/or mortality, and to determine if recommended interventions are having their expected population impact by examining discrepancies between controlled cancer intervention study results and the population experience.

Once a general understanding of the various factors influencing current trends has been achieved, a number of secondary goals may be addressed. Applicants may propose secondary goals of modeling the potential impact of new interventions on future national trends, and/or evaluating optimal cancer control strategies.

The NCI has a long-standing function of providing answers to critical policy questions, which can only be answered through an indirect synthesis of available information and assumptions. A commitment to modeling of this type will allow the NCI to apply the most sophisticated tools available for evidence-based planning to several areas: 1) Be responsive to challenges due to the increasing pace of technology, and to provide short-term answers while randomized controlled trials (RCTs) are still in progress. In the future we will be increasingly faced with new interventions, biomarkers, and diagnostic and genetic tests that will become widely disseminated prior to rigorous testing in controlled settings, and therefore the evaluation of population impact will become even more important. 2) Address emerging questions while they are still being debated in the policy forum. For example, new smokeless tobacco products are coming on the market, and modeling of their potential impact can benefit the Federal Trade Commission and other policy makers. 3) Translate RCT evidence of quantities to the population setting. 4) Provide estimates of quantities that will never be derived from RCTs. For example, half of Americans alive today who ever smoked are ex-smokers. It is important to understand the patterns of quitting, the process of carcinogenesis for ex-smokers, and the implications for future lung cancer trends.

DCCPS, which fulfills a federal-level function to respond to evolving surveillance questions of national policy relevance, helps focus research questions and acts as a conduit to national data resources necessary for parameter estimation, model calibration, validation, and population trends. An emergent property of this collaborative agreement is progress toward a comprehensive understanding of the determinants of site-specific cancer trends at the population level and a better understanding of the science of modeling.

Modeling is the use of mathematical and statistical techniques within a logical framework to integrate and synthesize known biological, epidemiological, clinical, behavioral, genetic, and economic information. Prior to the Cancer Intervention and Surveillance Modeling Network (CISNET), many of the simulation and other modeling techniques had been utilized to describe the impact of cancer interventions (i.e., primary prevention, screening, treatment) for hypothetical cohorts or in trial and other clinical settings. The goal of this request for applications (RFA) is to promote the application and extension of these models to population-based settings in order to ascertain determinants of cancer trends. This information is critical to the NCI because of the necessity of understanding whether recommended interventions are having their expected population impact, and of predicting the potential impact of new interventions on national trends. These studies will often involve extrapolation of results of controlled cancer intervention studies to estimates of U.S. population and community effectiveness. This type of modeling addresses issues of population-based policies and programs, and is distinct from individual-level models of risk and models of clinical decision making used at the individual patient–physician level. An additional goal of this concept is to advance methodology for modeling and to develop more uniform criteria for model validation in the population setting.

It is not the purpose of this RFA to focus on the analysis of hypothetical or trial-based cohorts and/or cost-effectiveness analyses, but rather to support analyses based on realistic scenarios of population impact. Projects will focus on models describing the population impact of the observed dissemination of cancer control interventions as well as other factors on observed national incidence and/or mortality trends.

CISNET was originally funded as a cooperative agreement (U01) for two phased-in rounds of funding. In September 2000, RFA CA-99-013 funded seven grants in breast cancer, one in prostate cancer, and one in colorectal cancer. A second round, funded under RFA CA-02-010 in August 2002, funded five grants in lung cancer as well as two additional grants for colorectal cancer and one in prostate cancer.

CISNET investigators are currently engaged in a wide range of policy-relevant modeling studies including the following:

1. *Development of base case questions.* A major strength of having a consortium of modelers is the ability to employ a comparative modeling approach. While each modeler has areas of individual focus, whenever possible, common “base” questions have been developed that allow for comparisons across models. The sometimes widely different results from models are often difficult to resolve, and base cases provide a chance to reach consensus on important questions, and to better understand differences between models. In these base case questions, a set of common population inputs is used across all models (e.g., dissemination patterns of screening and treatment, mortality from noncancer causes), and a common set of intermediate and final outputs is developed to help understand differences and similarities across models.
2. *Breast base case spin-off questions.* The breast base case serves as a jumping-off point for each grantee as they vary the basic formulation to focus on areas of individual interest. Spin-off issues that are actively being pursued include a) modeling the impact of using alternative, more biologically based natural disease history formulations, especially continuous time tumor growth models (which include microscopic fatal metastases that are initially undetectable); b) analyses for different racial, ethnic, and insurance-status groups; c) a unique Bayesian approach to update its prior estimates of treatment efficacy to obtain posterior estimates of community effectiveness of adjuvant therapy and mammography that best reproduce national mortality trends; d) geographically based analyses; e) the role of risk factors in breast cancer trends; and f) the potential impact of optimal screening intervals.
3. *Prostate cancer.* CISNET researchers have published an analysis of trends in the use of the prostate-specific antigen (PSA) test for modeling prostate cancer incidence trends to obtain estimates of over-diagnoses associated with PSA screening. In addition, these researchers are investigating the use of modeling to better understand the results of ecologic analyses of the effectiveness of PSA screening.
4. *Special issue of* Statistical Methods in Medical Research. CISNET was invited to sponsor a special issue of the journal *Statistical Methods in Medical Research* titled “Uses of Stochastic Models for the Early Detection of Cancer,” with articles submitted in spring 2003. Articles in the issue include 1) “Distribution of Clinical Covariates at Detection of Cancer: Stochastic Modeling and Statistical Inference,” 2) “Planning Public Health Programs and Scheduling: Breast Cancer,” 3) “Planning of Randomized Trials,” 4) “The Use of Modeling to Understand the Impact of Screening on U.S. Mortality: Examples from Mammography and PSA Screening,” 5) “Parameter Estimation for Stochastic Models via Simulation,” and 6) “Diversity of Model Approaches.”
5. *Linkages with other cancer surveillance and control activities.* CISNET has sought linkages to be integrated with and responsive to situations where modeling may play an important role. For example, the Agency for Health Research and Quality and the Center for Medicare and Medicaid Studies approached the NCI for assistance in studying a reimbursement decision related to the immunochemical fecal occult blood test (iFOBT) (http://cisnet.cancer.gov/reports/medicare.html). CISNET modelers have also been asked to aid in a midcourse (2005) evaluation to help determine whether reaching Healthy People 2010 upstream goals for cancer treatment, screening, and prevention will enable us to fall short of, meet, or exceed the downstream 2010 cancer mortality goals, and to retarget our efforts if necessary.

This reissuance of CISNET will be limited to modeling applications focusing on breast, prostate, lung, and colorectal cancer. Although the reissuance of CISNET will not be limited to grantees previously or currently funded, CISNET will no longer fund models that either are starting from scratch or have not been previously applied to the analysis of population trends. This means that models should have been applied to multiple real birth cohorts representing the actual population experience. Models that have been applied only to hypothetical cohorts, as is sometimes done to model trial data or estimate cost-effectiveness, will not be considered. The emphasis in this reissuance is in the application of already developed models to study the population impact of existing or emerging cancer control interventions. In addition, applications are being solicited for cancer site–specific coordinating centers for breast, prostate, colorectal, and lung cancer.

Areas of application will include more refined analyses of current trends, and a renewed emphasis on future trends and optimal cancer control planning. While the original issuance focused primarily on discovery (basic mathematical and statistical relationships necessary for the development of multi-cohort population models) and development (data sources and realistic scenarios to evaluate past intervention impact in the population setting and project future impact), the reissuance will continue development efforts and will greatly enhance the delivery element (synthesizing relevant scenarios for informing policy decisions and cancer control planning implementation).

While some new mathematical and statistical derivations may be necessary, they should not be the centerpiece of these applications. Instead, the focus of the application should be on identifying important cancer surveillance and control questions, obtaining the data sources and making model modifications as necessary to run the model, and producing results that are meaningful and packaged in a way that policy makers and cancer control planners can understand. Inclusion of interdisciplinary expertise will be essential in this phase of CISNET. Applicants should demonstrate modeling capability and propose a specific research plan. However, applicants should be flexible enough to accommodate further refinement and integration with other efforts.

The purpose of these efforts is to model the impact of the observed dissemination of cancer control interventions in the population, rather than using observed population trends to postulate new etiologic factors. However, these models can include components that model the impact of population changes in both modifiable and nonmodifiable risk factors. Models that include the synergistic impact of multiple interventions simultaneously are desirable. Models can be of the entire U.S. population, a region of the country, some specific identified population where unique data exist on the implementation of an intervention, or in a subpopulation of specific interest (e.g., the rural poor). However, whenever possible, inference should relate to the United States as a whole. Models can be developed for non-U.S. populations, but should be justified based on their applicability to understanding U.S. cancer trends.

Examples of areas of interest and types of questions are given below. Note that these are examples only, and applicants should not feel constrained to choose areas of application from this list only.

1. What new quantifiable statements can be made concerning estimates and uncertainty in the adenoma-colorectal cancer sequence? What is the range of natural history models associated with *in situ* breast cancer, and what are the implications of these natural history models for the overdiagnosis of disease?
2. What is the contribution of treatment to observed declines in prostate cancer mortality, especially the transition from the use of androgen deprivation therapy after biochemical failure (i.e., rising PSA levels) to use in the adjuvant setting? How can future improvements in the quality of care and the general health status of older individuals result in increased use and responsiveness to treatment?
3. What is the impact on incidence and mortality of both the increased dissemination of currently established screening modalities (e.g., iFOBT, sigmoidoscopy) and the potential dissemination of new or more novel modalities (e.g., screening colonoscopy, advanced imaging modalities, iFOBT, fecal mutagen tests, other innovative biomarkers)? As screening trial results for PSA, flexible sigmoidoscopy, chest X ray, and spiral computed tomography start to become available over the next decade, how do these results alter our understanding of population trends in incidence and mortality?
4. Given that obesity is a major problem that is getting worse, what are the implications for projections of breast and colorectal cancer mortality? What is the expected dissemination of the use of tamoxifen for women with different risk profiles, and what is the projected mortality reduction associated with these levels of dissemination?
5. How would resource requirements be affected by the use of risk stratification models or biomarkers that would allow selective screening and/or selective surveillance monitoring of higher-risk individuals? What is the national burden of iatrogenic morbidity from prostate cancer treatment among screen-detected men, and how do we weigh this against the potential mortality gains?
6. Can we use population trends to better understand differences in the natural history of prostate cancer between white and black men, and how can we use this information to better target interventions? How do racial disparities in obesity impact future trends? What is the impact of racial, economic, and insurance-status disparities in the use of adjuvant therapy and mammography on breast cancer mortality?
7. What is the impact of changing Medicare reimbursement policies on screening, treatment, and cancer mortality?
8. CISNET models should be able to help translate (in a timely manner) the impact of specific emergent results from epidemiologic, genetic, treatment, prevention, and screening studies to the population setting. Recent examples include how the mutation of a gene involved in non–small cell lung cancer increases the likelihood that the drug gefitinib will show a beneficial response; how a prevention trial showed that although finasteride reduced the risk of developing prostate cancer, those who developed the cancer had higher-grade tumors; and how an international clinical trial found that post-menopausal survivors of early-stage breast cancer who took the drug letrozole after completing an initial five years of tamoxifen therapy had a significantly reduced risk of cancer.
9. CISNET models can help translate the relationship between upstream (e.g., screening, modifiable risk factors) and downstream (e.g., mortality) goals. It can also help target the upstream factors that have the most potential for influencing mortality. In addition, CISNET models can help target what types of emerging technologies have the largest potential to help us reach the NCI’s 2015 goal of eliminating suffering from cancer. Is enough known about these technologies to have confidence in these projections? Can modeling point to the most important studies that could be conducted to gain more confidence with respect to their operating characteristics?

In the first issuance of CISNET no funds were specifically allocated for coordination activities. In this reissuance we have set aside funds for coordinating centers for all four cancer sites: breast, prostate, colorectal, and lung cancer. Coordinating centers should be site-specific because each center needs to be totally conversant with the data sources, modeling issues, and policy questions specific to that cancer site. Coordination activities, under the general direction and consensus of the NCI and principal investigators, will include 1) formulating, prioritizing, and coordinating work on base case and other questions (including outside requests); 2) negotiating common requests for outside data sources; 3) consensus building and coordinating critical evaluation of disparate results; 4) preparing inputs, and collecting and processing common outputs for model comparisons; and 5) coordinating synthesis papers and group responses, bringing together disparate information to inform policy makers. Through the coordinating center, each CISNET cancer site group will constitute an established expert knowledge base that can provide technical advice on evolving policy-relevant surveillance questions. Because all of the expertise necessary to accomplish these goals is not likely to exist in one place, the coordinating center would have discretionary funds to tap outside expertise for particular tasks, pay for access to data sources, and provide funds to modeling groups to mount intensive efforts to provide technical advice while issues are still relevant in the policy area. Even though one group would be tasked with being the coordinating center, CISNET would be run through consensus, as it has in the past.

To keep applications focused, each will be limited to a single cancer site. The CISNET project requirements call for the development of site-specific working groups that will 1) facilitate comparative analyses, 2) allow modeling groups access to a broader array of data resources and interdisciplinary expertise, and 3) provide a forum for discussions of validation and other methodologic issues. CISNET will allow for diversity and originality of modeling approaches that can be compared using uniform criteria. New investigators will be expected to join in the ongoing collaborative activities already under way.

The NCI intends to commit approximately $1.8 million in total costs (direct and facilities and administrative [F&A] costs) in fiscal year 2005 to fund 6–9 new modeling grants in response to this RFA. In addition, the NCI intends to commit approximately $950,000 (direct and F&A costs) in fiscal year 2005 to fund 4 coordinating centers (one each in breast, prostate, colorectal, and lung cancer). An applicant may request a project period of up to five years. Although an applicant can submit applications for more than one cancer site (either for modeling grants or coordinating centers), each individual application must be limited to one cancer site. Coordinating center grants must be submitted separately from modeling grants, even if one applicant submits both.

Applications must be prepared using the PHS 398 research grant application instructions and forms (rev. 5/2001). Applications must have a Dun and Bradstreet Data Universal Numbering System (DUNS) number as the Universal Identifier when applying for federal grants or cooperative agreements. The DUNS number can be obtained by calling 1-866-705-5711 or through the website at http://www.dunandbradstreet.com/. The DUNS number should be entered on line 11 of the face page of the PHS 398 form. The PHS 398 document is available at http://grants.nih.gov/grants/funding/phs398/phs398.html in an interactive format. For further assistance, contact GrantsInfo by calling 301-435-0714 or e-mailing GrantsInfo@nih.gov.

Letters of intent must be received by 14 September 2004. Applications must be received by 14 October 2004. The anticipated award date is July 2005.

Contact: Eric Feuer, DCCPS, NCI, 6116 Executive Blvd, Rm 5041, MSC 8317, Bethesda, MD 20892-8317 USA, 301-496-5029, fax: 301-480-2046, e-mail: rf41u@nih.gov. Reference: RFA No. RFA-CA-05-018

## Defender of the Earth Book Award

Red Hen Press announces the first annual Defender of the Earth Book Award to promote awareness and appreciation of the need to respect the beauty and fragility of the environment. This award is for a previously unpublished original manuscript of nonfiction writing on the environment, and is open to all authors. The award is to be presented by the David Family Foundation.

The winner will receive $5,000 and publication of the winning manuscript by Red Hen Press. The minimum page count for submissions is 64 pages. The author’s name should appear only on the cover sheet. Send a self-addressed stamped envelope for notification. Entries must be postmarked by 30 August 2004.

Contact: Red Hen Press, Attention: Defender of the Earth Book Award, PO Box 3537, Granada Hills, CA 91394 USA, 818-831-0649, e-mail: editors@redhen.org, Internet: http://www.redhen.org/2004literaryawards.htm

